# Soil Ecotoxicology Needs Robust Biomarkers: A Meta‐Analysis Approach to Test the Robustness of Gene Expression‐Based Biomarkers for Measuring Chemical Exposure Effects in Soil Invertebrates

**DOI:** 10.1002/etc.5402

**Published:** 2022-08-03

**Authors:** Elmer Swart, Ellie Martell, Claus Svendsen, David J. Spurgeon

**Affiliations:** ^1^ UK Centre for Ecology and Hydrology Wallingford UK; ^2^ United Kingdom Department for Environment Food & Rural Affairs London UK

**Keywords:** Biomarker, Soil invertebrate, Meta‐analysis, Gene expression, Metallothionein, Heat shock protein

## Abstract

Gene expression‐based biomarkers are regularly proposed as rapid, sensitive, and mechanistically informative tools to identify whether soil invertebrates experience adverse effects due to chemical exposure. However, before biomarkers could be deployed within diagnostic studies, systematic evidence of the robustness of such biomarkers to detect effects is needed. In our study, we present an approach for conducting a meta‐analysis of the robustness of gene expression‐based biomarkers in soil invertebrates. The approach was developed and trialed for two measurements of gene expression commonly proposed as biomarkers in soil ecotoxicology: earthworm metallothionein (MT) gene expression for metals and earthworm heat shock protein 70 (HSP70) gene expression for organic chemicals. We collected 294 unique gene expression data points from the literature and used linear mixed‐effect models to assess concentration, exposure duration, and species effects on the quantified response. The meta‐analysis showed that the expression of earthworm MT was strongly metal concentration dependent, stable over time and species independent. The metal concentration‐dependent response was strongest for cadmium, indicating that this gene is a suitable biomarker for this metal. For copper, no clear concentration‐dependent response of MT gene expression in earthworms was found, indicating MT is not a reliable biomarker for this metal. For HSP70, overall marginal up‐regulation and lack of a concentration‐dependent response indicated that this gene is not suitable as a biomarker for organic pollutant effects in earthworms. The present study demonstrates how meta‐analysis can be used to assess the status of biomarkers. We encourage colleagues to apply this open‐access approach to other biomarkers, as such quantitative assessment is a prerequisite to ensuring that the suitability and limitations of proposed biomarkers are known and stated. *Environ Toxicol Chem* 2022;41:2124–2138. © 2022 The Authors. *Environmental Toxicology and Chemistry* published by Wiley Periodicals LLC on behalf of SETAC.

## INTRODUCTION

Soil communities can be exposed to metals, polycyclic aromatic hydrocarbons (PAHs), human and veterinary pharmaceuticals, and other industrial and consumer product‐associated chemicals. The presence of these chemicals can potentially cause adverse effects on exposed populations (Eom et al., 2007; Mirmonsef et al., 2017; Spurgeon & Hopkin, 1996). Measurements of pollutant concentrations through chemical analysis is a key way in which soil contamination can be identified. However, chemical analysis alone cannot provide a complete picture of the bioavailability of chemicals or the potential mixture effects which may drive toxicity in the environment (Cedergreen, 2014; Kortenkamp & Faust, 2018). To provide additional evidence of the biological consequences of pollutant exposure in soil species, many authors of studies over at least a quarter of a century (if not longer) have recommended the use of biological measurements, commonly known as biomarkers, as an additional means to identify chemical exposure and effects on species across a range of monitoring (Brack et al., 2016; Colin et al., 2016) and high‐throughput toxicity screening (e.g., ToxCast) applications (Richard et al., 2016).

Over the years, numerous individual studies have identified different biomarkers in different species‐chemical pair studies. However, if such measurements are ever truly to be used in diagnostic applications, then systematic evidence of the robustness and relevant application domains of the method that goes beyond that possible within any individual study is needed. As the number of biomarker studies increases, it becomes feasible to apply quantitative evidence review methods to assess the utility of different biomarkers for pollutant assessment. Previous studies have applied evidence review methods to questions in ecotoxicology, such as on the nature of microplastic pollution, the relative toxicity of nano versus ionic forms of metals for aquatic species, the relative sensitivity of earthworm species to pesticides, and the effect of earthworms on organic chemical biotransformation (Burns et al., 2018; Chao et al., 2022; Notter et al., 2014; Pelosi et al., 2013). However, to date there has been only a limited application of evidence review methods to assess biomarker suitability. One such study by Shahid (2021) was able to use evidence review to assess how soil amendment influences trace element‐induced oxidative stress‐related parameters in plants. This application indicates that such approaches can be applied for this kind of suborganism data.

To demonstrate how evidence review methods can be used to verify and direct the use of biomarkers to study chemical exposure and effects in soil ecosystems, we conducted a meta‐analysis to assess the robustness and suitability of proposed gene expression‐based biomarkers. Gene expression measurements are considered as rapid and sensitive tools able to provide mechanistic information about toxic effects (Nota et al., 2008; Spurgeon, Ricketts, et al., 2005; Svendsen et al., 2008; Van Straalen & Roelofs, 2008). The earliest gene expression‐based biomarkers in soil organisms were developed to measure metal exposure effects in Lumbricidae earthworms using gene expression of metallothionein (MT), a protein involved in metal binding and transport, as a biomarker (Stürzenbaum et al., 1998). Since then, many more potential gene expression‐based biomarkers have been identified and studied for their responses to chemical exposure in both the laboratory and the field in various soil species including various Lumbricidae earthworms, springtails and enchytraeids (Brulle et al., 2007; Nota et al., 2008; Novais et al., 2012; Pérès et al., 2011). Increasingly, next‐generation sequencing approaches, such as transcriptomics, have been used to identify new biomarkers and to quantify their responses to chemical stressors (Gong & Perkins, 2016; Roelofs et al., 2012; Simões et al., 2018). Through these efforts a large set of biomarkers has been tested for use in individual studies. However, a systematic analysis of how suitable these biomarkers are to measure chemical exposure effects in soil invertebrates is currently missing, meaning that information on the robustness of these biomarkers for assessment across different cases is currently not available.

In the present study, we use quantitative meta‐analysis to study the applicability and robustness of gene expression‐based biomarkers for assessing the effects of chemical exposure on soil organisms. In conducting this analysis, we did not intend to fully elucidate the biological function of the specific biomarkers or to fully understand the nature of gene expression change in relation to specific chemical exposures. Such assessments would require a different and more integrated set of mechanistic toxicology studies across different chemical and species, including the use of comprehensive transcriptomic (e.g., RNA sequencing [RNAseq]) methods. Instead, we aimed to indicate how evidence review can be used to investigate biomarker robustness and application based on existing published data that may differ in its nature and quality. Within this remit, we specifically addressed the following two research questions: 1) Which are the most used gene expression‐based biomarkers for metal and organic pollutants in soil organisms? And 2) How responsive and robust are these biomarkers in their response as measures for chemical exposure and effects?

To assess robustness, the performance of gene expression‐based biomarkers was assessed against a set of previously defined criteria (Amiard‐Triquet & Amiard, 2013; Forbes et al., 2006; McCarthy John & Shugart, 1990). First, a good biomarker needs to show a positive concentration‐dependent response. Second, the gene expression of a biomarker should be relatively stable over time. Third, biomarker responses should (ideally) be relatively universal for a species group. Fourth, biomarkers should be specific to (a group of) chemicals. Finally, a biomarker response should also be indicative for adverse effects at organism or population level. These criteria, however, do not necessarily apply to all biomarkers equally and different weights to these criteria may be given depending on the research needs and the study system.

To address the research questions, we extracted metadata from a total of 83 peer‐reviewed publications that investigate the gene expression response of soil invertebrate species to chemical exposures published between 2003 and 2021. We collected in total 294 gene expression data points that were used in a meta‐analysis to test the suitability of the identified biomarkers according to the identified criteria. By following existing guidelines for quantitative evidence review, we provide an informed conclusion on the current state and future needs of the use of gene expression‐based biomarkers in soil invertebrates. All raw data and R scripts used for the analysis are openly available under CC BY 4.0 at https://doi.org/10.5281/zenodo.5145029 (Swart et al., 2022). Therefore, the present study may help to guide future biomarker development in soil ecotoxicology. We strongly encourage colleagues in the field to conduct similar assessments for other biomarkers.

## METHODS

### Question 1: Which are the most used gene expression‐based biomarkers for metal and organic pollutants in soil organisms?

#### Literature search

Quantitative meta‐analyses aim to provide an informed and unbiased conclusion on the size and characteristics of an evidence base. To ensure robustness and transparency, the literature review and analysis procedure was determined prior to the start of the investigation following guidelines for rapid evidence reviews provided by the UK Department for Environment, Food and Rural Affairs (Collins et al., 2015). There were no specific geographical restrictions for this review. However, only peer‐reviewed literature published in English was considered, but no date restrictions were applied. This review only assessed the use of biomarkers based on the mRNA expression of marker genes (“gene expression” herein). Studies using methods to assess epigenetics, protein expression or nontargeted metabolite profiles were not considered with the assessment due to the small evidence size of these methods which precluded the application of quantitative meta‐analysis approaches. To identify publications relevant to our research questions, we defined four additional criteria that were used to select papers for the final evidence extraction:

1. *Chemical pollutants*: Chemicals included in this review should either be used as pesticides in agriculture or be likely to end up as contaminants of agricultural soils through, for example, the application of sludge or air depositions. Publications that assessed effects of organic of mineral fertilizers or nitrogen depositions were not considered.
2. *Soil*: Only studies conducted in soils should be considered. This excludes studies that used soil organisms in mediums other than soil (e.g., liquid medium) and studies conducted in sediments or sludge material prior to being applied to land.
3. *Soil invertebrates*: Only studies relating to invertebrates living in or on soils for the larger part of their life‐cycle were considered. Above ground terrestrial species (e.g., pollinators) were not included.
4. *Gene expression methods*: Considered approaches were restricted to the following omics techniques: quantitative polymerase chain reaction (PCR), RNAseq and micro‐array‐based transcriptomics.

A set of four search term populations were identified, see Table 1. Using these populations and Boolean operators, a literature search was conducted using two search systems: Web of Science Core collection and the PubMed database. Searching for literature in more than one search system aimed to widen the search and thereby increase the potential evidence size. The literature search was conducted on February, 10 2021. Metadata of all identified publications were exported to MS Excel and duplications (i.e., publications identified in both search systems) removed. After analysis of the collected metadata, two additional specific literature searches were conducted to identify additional publications relevant for the second research question. The search terms used in this additional search are shown in Table 2.

**Table 1:** Search terms in combination with Boolean operators (in italics) used in the Web of Science core collection and PubMed database to identify potentially relevant publications

Chemical pollutants (*AND*)	Soil (*AND*)	Soil invertebrates (*AND*)	Gene expression methods (*AND*)
pesticide	soil	invertebrate	*genomic*
herbicide		insect	transcripto*
fungicide		collembol*	gene expression
insecticide		springtail	microarray
chemical		enchytr*	RNA sequencing
pollution		mite	
metal		nematode	
nano*		earthworm snail	

**Table 2:** Search terms used in the Web of Science core collection to identify additional publications relevant for the second research question

Search focus summary	Search term
Metallothionein gene expression in earthworms exposed to metals	TS = (earthworm) AND TS = (metal OR copper OR zinc OR cadmium OR silver) AND TS = (metallothionein) and TS = (gene expression)
Heat shock protein gene expression in earthworms exposured to (organic) pollutants	TS = (earthworm) AND TS = (pesticide OR herbicide OR fungicide OR insecticide OR chemical OR pollution) AND TS = (heat shock protein OR HSP) AND TS = (gene expression)

#### Relevance screening

Potential papers were screened for relevance to the research questions in two phases. In the first screen, studies were categorized as “not relevant,” “relevant,” or “uncertain” based on the title of the publication. In the second phase, all publications that were scored as “relevant” or “uncertain” were assessed in detail for applicability based on the abstract of the whole document of the publication (see Supporting Information, Table S3.1). All publications categorized as “relevant” after the second screening phase were then taken further for metadata collection.

#### Metadata collection

Metadata from each relevant publication were collected in MS Excel. The collected data (in total 38 variables) included information on the publication (e.g., publication year, title and a unique publication identifier), study design (e.g., study type, primary exposure chemical, soil type, invertebrate species, exposure concentration and duration, gene expression measurement method), studied biomarkers (expression of MT, catalase, heat shock protein, etc.), and whether the publication was suitable for data extraction of gene‐specific expression values. For the variable “study type,” studies were categorized as one of four main types: 1) “spiked experimental studies” (studies that exposed naïve populations of invertebrates to soils that are spiked in the laboratory), 2) “polluted field soil experimental studies” (studies that exposed naïve populations in the laboratory to soils collected from chemically polluted fields), 3) “in situ studies” (studies that measured the in situ gene expression of biomarkers in invertebrates collected from polluted fields), and 4) “ex situ studies” (studies that collected invertebrates from polluted field populations and used these in laboratory experiments). Many publications contained gene expression data for more than one chemical or more than one invertebrate species. In this review, we distinguish “publications” from “studies.” The former refers to the actual report published in a scientific journal, the later to a unique combination of a chemical and a soil organism used for biomarker analysis. Hence, a single “publication” can contain multiple “studies.” All collected metadata and an explanation of each variable can be found in Supporting Information, Table S3.2.

### Question 2: How responsive and robust are these biomarkers in their response as measures for chemical exposure and effects?

#### Gene expression data collection

Not all identified studies were appropriate for gene expression data collection. Some studies reported median but not mean values, others only reported the model statistics of biomarker responses or showed gene expression profiles in a form that did not allow for extraction of the mean values (e.g., heatmaps or pathway analysis as typically used in studies using RNAseq). Furthermore, studies that used organisms collected from contaminated fields were excluded (i.e., in situ and ex situ studies, see above paragraph for a definition of these terms). These populations may have undergone genetic adaptations to pollution which could affect the expression of biomarkers and thereby confound our analysis (Roelofs et al., 2009; Timmermans et al., 2005). Furthermore, the exposure history of these organisms may not be known, which again may affect gene expression.

The analysis of the metadata showed that the best‐studied species biomarkers for metals and organic pollutants were respectively metallothionein (in the literature referred to as cadmium‐metallothionein or metallothionein isoform 2, but hereafter metallothionein or MT) and heat shock protein 70 (HSP70) expression in earthworms (see *Results* section). All publications that used these two species biomarkers and contained extractable gene expression data were used for a further round of data extraction. In this second stage, fold changes between the mean gene expression in treatment and controls were collected from tables or from figures using the web‐based tool WebPlotDigitizer (Rohatgi, 2020). In cases where fold changes were not reported, fold changes were calculated from absolute gene expression values. The fold change measurements were then log2 transformed to center the gene expression data on zero to give equal weight to up‐ and down‐regulation. This specific gene expression data can be found in Supporting Information, Table S3.3. For the HSP70 data, an extra variable indicating the mechanism of action of each organic chemical was defined based on best available information. This was done to allow for grouping of chemicals and to account for differences in gene expression profiles between chemicals with different mechanisms.

#### Statistical analysis

We conducted linear mixed‐effect modelling using the R package “lmeTest” (Kuznetsova et al., 2017) to test for the impact of the explanatory variables on gene expression (see Supporting Information, Table S1, for a list of used variables). In all models, the publication identifier was added as a random effect to account for the fact that multiple data points came from single studies. MT and HSP70 data were analysed separately. In both analyses, we first identified which interactions between the explanatory variables were important to include in the mixed‐effect model. Interactions with either “exposure duration” or “exposure concentration” became a focus, as these two variables were expected to be the main drivers of gene expression based on general understanding of stress responses. All explanatory variables and significant interactions were then included in a global model (Table 3). To assess for overall model significance, models were tested against a reduced model which only included publication identifier as random effect and used the maximum likelihood approach for fitting. However, all model estimates were derived using the restricted maximum likelihood approach, which is the best approach for mixed‐effect models. Testing for the significance of the main effects was done using the “anova” function applying a type III analysis of variance table and Satterthwaite's method for calculation of degrees of freedom. The statistical analysis of the global HSP70 model indicated that the only significant term was the interaction between exposure duration and mechanism of action. In cases of weak relationships, the inclusion of multiple interaction terms can hide the effects of main effect variables. Therefore, for the HSP70 analysis we in addition conducted an automated model selection procedure based on Akaike Information Criterion (AIC) using the “MuMIN package” (Barton, 2020) to identify the “best model”, which was subsequently tested for significance of effects using the same approach as for the other models.

**Table 3:** Composition of linear mixed‐effect models used to test gene expression patterns

Gene	Type	Dependent variable	Explanatory variables (fixed effect)^a^	Random effect
MT	Reduced	Log2FC_MT_	(None)	Publication
MT	Global	Log2FC_MT_	Concentration + Exposure duration + Species + Metal + Study type + Exposure duration:Metal	Publication
HSP	Reduced	Log2FC_HSP_	(None)	Publication
HSP	Global	Log2FC_HSP_	Concentration + Exposure duration + Mechanism of action + Exposure duration:Concentration + Exposure duration:Mechanism of action	Publication
HSP	Best	Log2FC_HSP_	Concentration + Exposure duration + Mechanism of action + Exposure duration:Mechanism of action	Publication

^a^ Explanatory variables separated by colon are interaction effects.

Log2FC_MT_ = log2 fold change of the gene expression of metallothionein (MT) in earthworms; Log2FC_HSP_ = log2 fold change of the gene expression of heat shock protein in earthworms.

## RESULTS

### Search and screening overview

**Figure 1:**
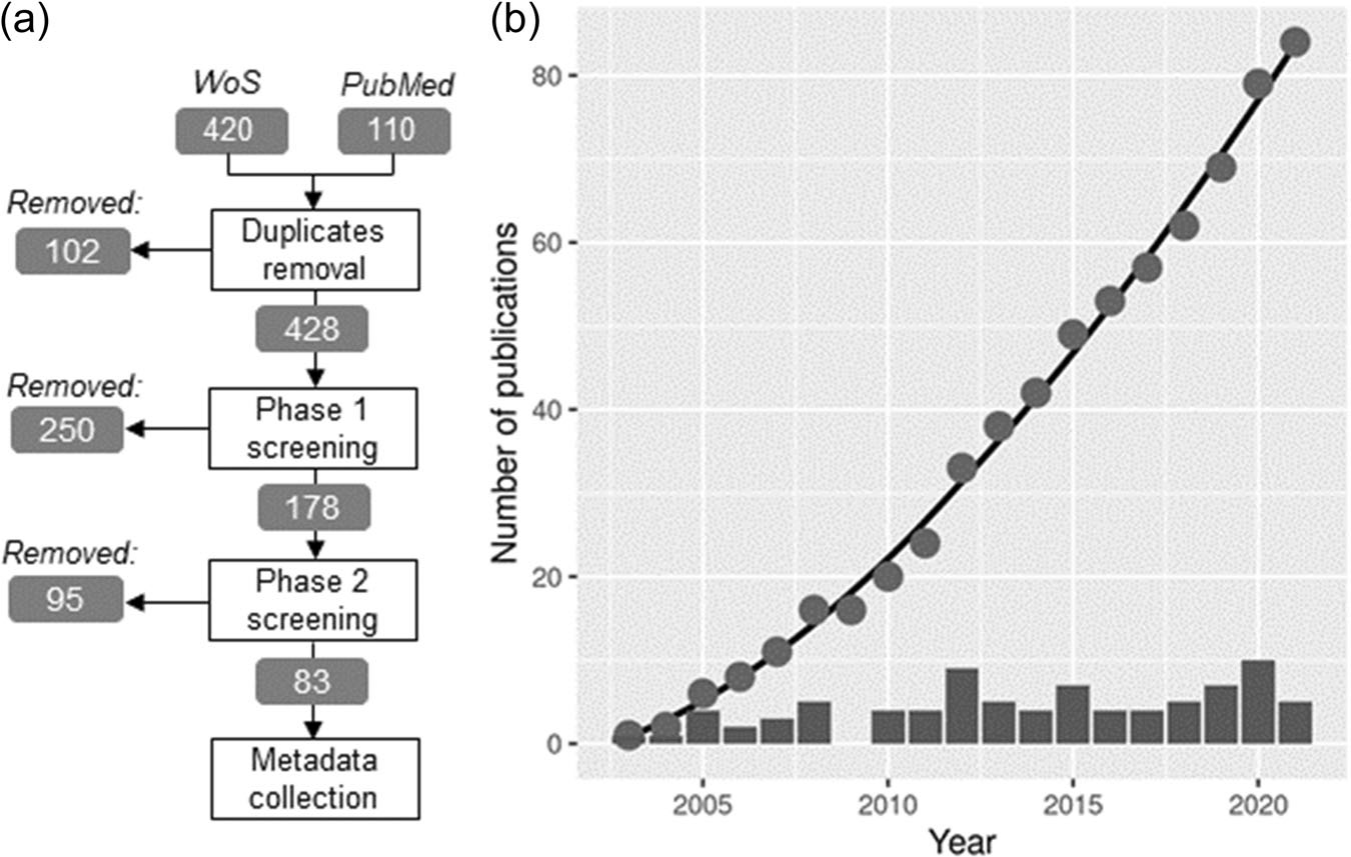
Overview of the evidence collection methods and results. (**A**) Evidence identification map with the numbers of publications remaining and removed at each stage. (**B**) The total cumulative number of relevant publications per year (closed circles) and the number of relevant papers per year (bars). WoS, Web of Science.

The literature search identified in total 428 publications that were potentially relevant for the research after the removal of duplicates (Figure 1A). Screening based on the publication title (Phase I screening) removed 250 of these publications. By assessment of the publication abstract or the whole document (Phase II screening), we identified in total 83 relevant publications that used transcriptomics methods to investigate species responses to pollution. The earliest transcriptomics‐based species biomarker studies were published in 2003. The average number of publications published between 2016 and 2020 was six, showing that functional gene expression‐based species biomarkers continue to be a topic of research in environmental science, although interest has neither grown nor declined since the original publication (Figure 1B).

### Question 1: Which are the most used gene expression‐based biomarkers for metal and organic pollutants in soil organisms?

**Figure 2:**
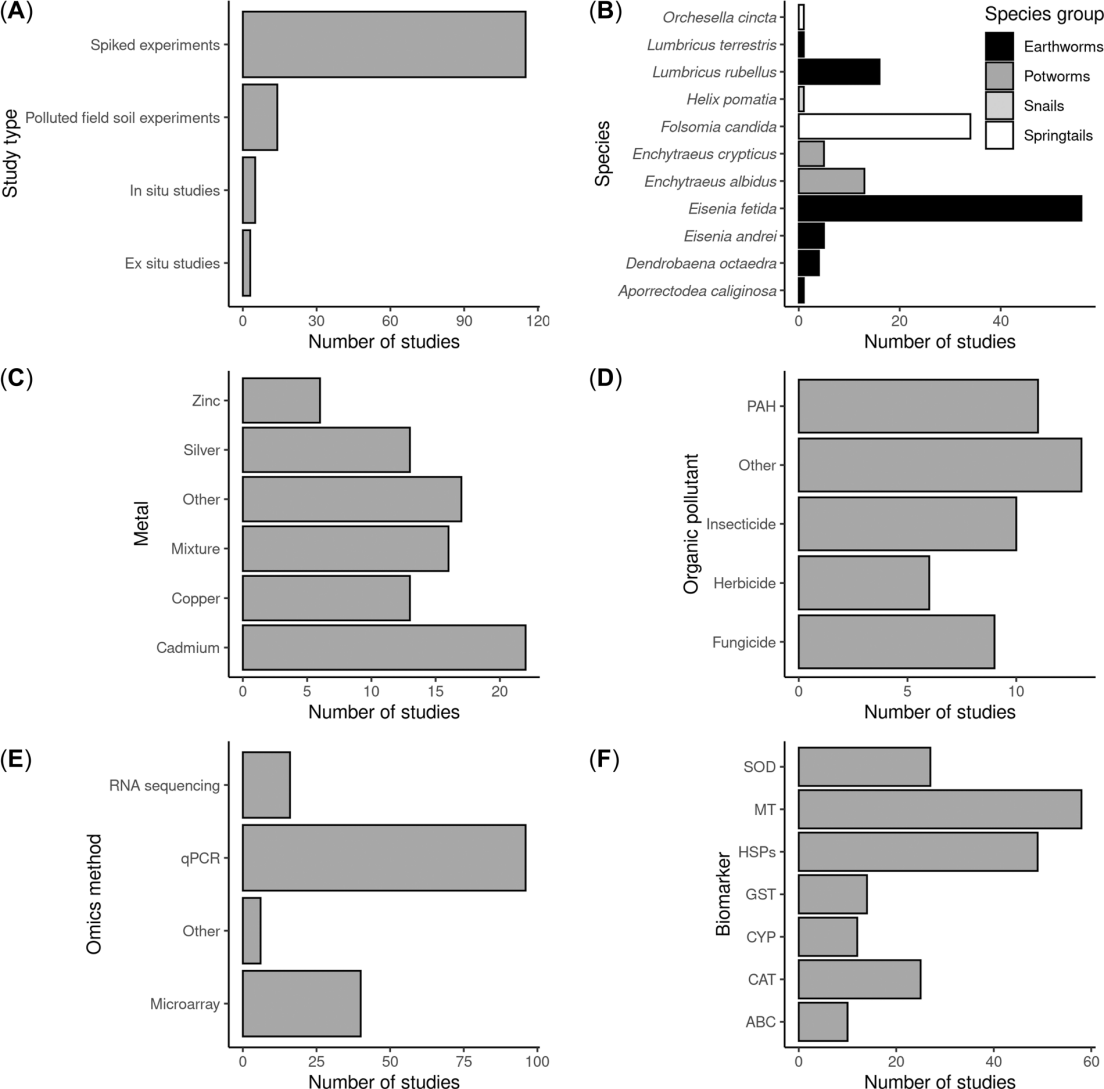
Overview of the composition of the species biomarker dataset showing the number of studies per (**A**) study type, (**B**) species and species group, (**C**) metal, (**D**) organic pollutant class, (**E**) omics method used for expression measureent, and (**F**) biomarker. For heat shock proteins (HSPs), the number shown is the total number of studies that use any class of HSP (e.g., HSP70, HSP90, etc.). ABC, ABC transporters; CAT, catalase; CYP, cytochrome p450; GST, glutathione‐*S*‐transferase; HSP70, heat shock protein 70; MT, metallothionein; PAH, polycyclic aromatic hydrocarbon; qPCR, quantitative polymerase chain reaction; SOD, superoxide dismutase.

We extracted information on 38 qualitative variables from a total of 137 studies from 83 publications (see *Metadata collection* in the *Methods* section for a definition of these terms). A large majority of the studies (129 out of 137) were experimental studies which mostly used soils spiked in the laboratory (115) and only a small number (14) used polluted soils collected from fields (Figure 2A). Only eight studies used animals collected from polluted fields (i.e., in situ and ex situ studies).

Earthworms belonging to the family Lumbricidae (hereafter, “earthworms”) were by far the most used species group (96 studies), followed by springtails (35), earthworms from the family Enchytraeid (hereafter, “potworms”; 18), and snails (1; Figure 2B). Approximately two‐thirds of the studies assessed the effects of inorganic chemicals on gene expression (87), with the remainder studying the effects of organic chemicals (49). Cadmium was the most‐studied metal (22), followed by copper and silver (both 13; Figure 2C). Among studies that assessed the effects of organic chemicals on invertebrate gene expression, most focussed on synthetic pesticides (28 in total), and of these most focussed on insecticides (10), followed by fungicides (9) and herbicides (6; Figure 2D). Eleven studies investigated the effects of PAHs on gene expression biomarkers.

For the measurement of the biomarker effects in exposed organisms, most studies (70%) used quantitative PCR, whereas 29% and 12% of the studies used microarray and RNAseq, respectively (Figure 2E). Measurement of mRNA expression for MT was the most tested biomarker (58 studies), followed by heat shock protein genes (49), superoxide dismutase (27), and catalase (25; Figure 2F). Most studies used an artificial soil as the medium for the experimental studies (e.g., Organisation of Co‐operation and Development [OECD] 207; 46 studies), recognizing its role as the recommended test media for regulatory soil invertebrate toxicity testing. Many studies (29) did not report basal soil characteristics such as soil texture. The most‐tested natural soil was the agricultural loamy sand soil LUFA2.2 (35), reflecting that this soil is widely used as an alternative to artificial soil for toxicity testing.

**Figure 3:**
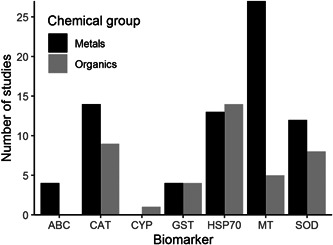
Number of relevant experimental studies that used earthworms per biomarker and chemical group. ABC = ABC transporters; CAT = catalase; CYP = cytochrome p450; GST = glutathione‐*s*‐transferase; HSP70 = heat shock protein 70; MT = metallothionein; SOD = superoxide dismutase.

Not all studies that used transcriptomic tools to investigate species responses to chemical pollution reported the data in such a way that the expression level of individual genes could be extracted. Of the 137 available studies, 71 provided relevant gene expression data for the quantitative biomarker analysis. Of these, most were for effects measured in earthworms (50 studies), followed by springtails (15), potworms (5), and snails (1). Forty‐two of these studies used metals and 29 used organic chemicals. We sought to identify the current best‐studied biomarkers for both metal and organic pollutants. Due to the limited number of relevant studies for springtails, potworms, and snails, we focussed this analysis on studies that used earthworms only. Among studies with earthworms that contained extractable gene expression information, MT expression was by far the most‐studied biomarker for metal pollutants and HSP70 expression was the most‐studied biomarker for organic pollutants (Figure 3). These two species biomarkers were selected for further gene expression data extraction using both publications identified from the first search and a further eight papers not identified in the initial general search, but found using refined search terms (see Table 2 and *Literature search* in the *Methods* section).

### Question 2: How responsive and robust are these biomarkers in their response as measures for chemical exposure and effects?

The analysis of the metadata (see Figure 3) showed that the best species biomarker for metal pollutants was MT expression in earthworms. The best‐studied species biomarker for organic pollutants was HSP70. To assess how responsive and robust these biomarkers are in their response as a measure for chemical exposure and effects, a quantitative meta‐analysis was conducted (see *Gene expression data collection* and *Statistical analysis* in the *Methods* section for details of this analysis).

#### MT expression in earthworms exposed to metals

In total, 203 data points relating to MT expression in earthworms under metal exposure were extracted from the published papers on this topic. This gene expression data was analysed using linear mixed‐effect models. First, we identified which interactions between the explanatory variables were important to include in the mixed‐effect model. This was done to reduce the complexity of the global model. In the present study, we only tested for interactions with either “exposure duration” or “exposure concentration” and the other explanatory variables (i.e., study type, species and metal), as these two variables were expected to be the main drivers of gene expression based on general understanding of stress responses. Only one interaction was significant (Supporting Information, Table S2; i.e., exposure duration and metal) and therefore only this interaction term was included in the global model.

**Figure 4:**
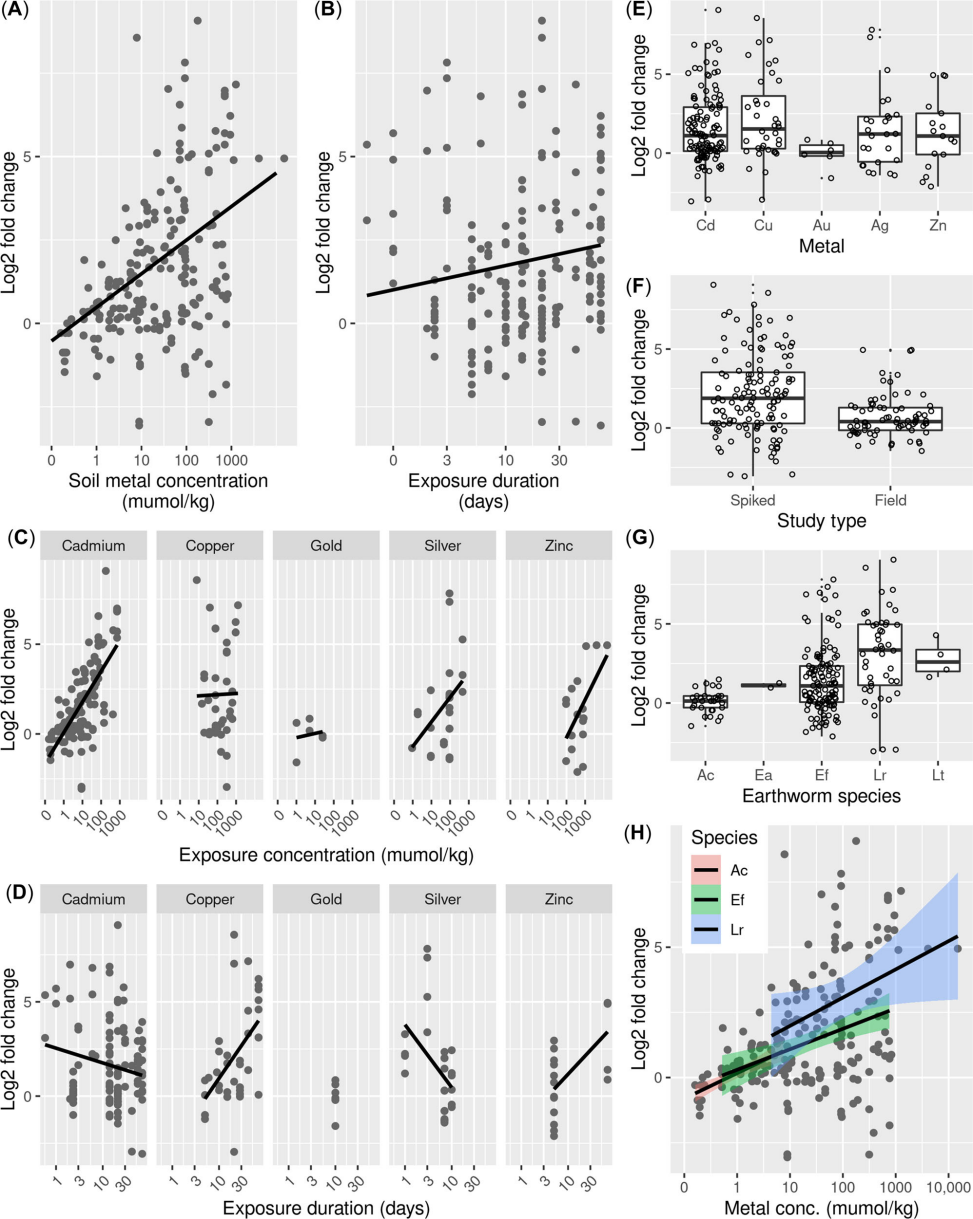
Relationship between log2 fold change of metallothionein (MT) gene expression in earthworms and (**A**) soil metal concentration, (**B**) exposure duration, (**C**) soil metal concentration per metal, (**D**) exposure duration per metal, (**E**) metal, (**F**) study type, (**G**) earthworm species, and (**H**) soil metal concentration per species. In (**E**)–(**H**), open circles represent data points, and lower and upper boxes correspond to the 25th and 75th percentiles with the upper/lower whiskers extend from the box to the highest/smallest value at most 1.5 × interquartile range of the box. Note that the different data points in the graphs are not all independent from each other but nested in “Publication.” Therefore, patterns (or in some cases the lack of) that can be observed through visual inspection do not always match statistical results. In (**F**): spiked, spiked experiments; field, polluted field soil experiments. In (**G**)–(**H**): Ac = *Aporrectodea caliginosa*; Ea = *Eisenia andrei*; Ef = *Eisenia fetida*; Lr = *Lumbricus rubellus*; Lt = *Lumbricus terrestris*.

The global MT model (Table 3) predicted the gene expression data significantly better than the reduced model, *χ* ^2^(14) = 65.954, *p* < 0.001. The publication identifier (which was added as a random effect) explained 49% of the total variance. Within the model, soil metal concentration had a strong positive effect on the expression of MT, but this effect was not significantly metal dependent (i.e., there was no significant interaction effect between concentration and metal, see Supporting Information, Table S2; Figure 4A,C and Table 4). Exposure duration also had a small, but significant, positive effect on MT gene expression (Figure 4B). However, there was also an interaction between exposure duration and chemical group, as can be observed by the different slopes of the linear models in Figure 4D (Table 4). MT gene expression was metal dependent (i.e., the model term “metal” had an significant impact on the explanatory variable), with copper having a strong negative effect on MT expression (Table 4 and Figure 4E). The model selection outcome indicated that the earthworm species and study type (studies that used soils spiked in the laboratory vs. soils collected from polluted fields) did not significantly affect measured gene expression (Figure 4E–F). Indeed, the slopes of the relationship between MT gene expression and the main explanatory variable (i.e., metal soil concentration) were similar in different earthworm species, indicating that metal‐induced MT gene expression is not species specific between different earthworms (Figure 4H).

**Table 4:** Statistics of the mixed‐effect model that best explained the earthworm metallothionein gene expression data

	Dependent variable: Log2FC_MT_		
	Slope estimate	SE	*p* value
Constant	−1.356	1.983	0.502
Concentration	1.008	0.218	<0.001***
Exposure duration	0.727	0.374	0.015*
Metal			0.004**
Copper	−8.604	2.293	
Gold	−2.075	1.339	
Silver	0.902	1.504	
Zinc	4.011	3.169	
Species			0.979
*Eisenia fetida*	1.383	2.836	
*Eisenia andrei*	0.621	1.831	
*Lumbricus rubellus*	0.490	1.978	
*Lumbricus terrestris*	−0.004	2.325	
Study type			0.682
Polluted field soil experiments	−0.441	1.070	
Exposure duration:Metal			<0.001***
Copper	6.196	1.407	
Silver	−2.577	1.269	
Zinc	1.644	2.481	
Observations	203		
Log likelihood	−388.95		
Akaike Information Criterion	811.89		
Bayesian Information Criterion	868.22		

Log2FC_MT_ = log2 fold change of the gene expression of metallothionein in earthworms; MT = metallothionein; SE = standard error.

*p < 0.05; **p < 0.01; ***p < 0.001.

#### HSP70 expression in earthworms exposed to organic pollutants

In total 91 data points relating to HSP70 expression in earthworms were collected from the literature. This data set was analysed using linear mixed‐effect models in which publication identifier was added as a random effect. The global model, which included all explanatory variables and interaction effects, significantly explained the HSP70 gene expression data better than the reduced model (*χ* ^2^(12) = 36.254, *p* < 0.001). In the global model, the publication identifier explained 18% of the total variance. However, according to this global model, the only significant term was the interaction between exposure duration and mechanism of action. In cases of weak relationships, the inclusion of multiple interaction terms can hide the effects of main effect variables. Therefore, for the HSP70 analysis we in addition conducted an automated model selection procedure based on AIC.

**Figure 5:**
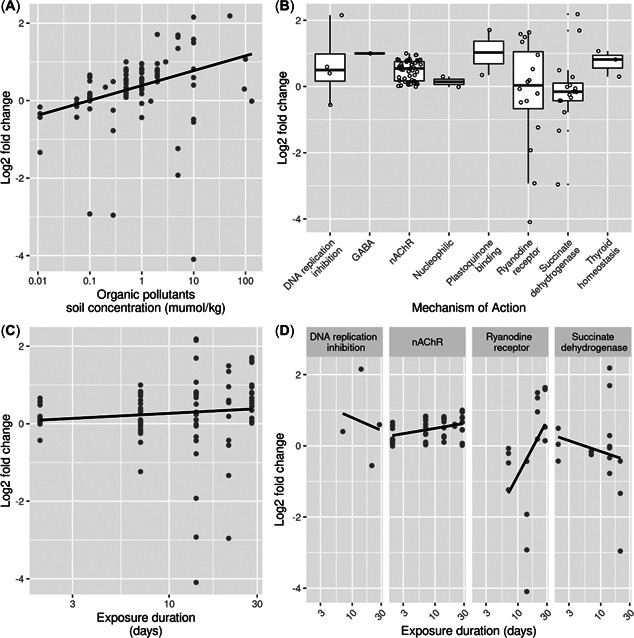
Relationship between log2 fold change of heat shock protein 70 (HSP70) gene expression in earthworms and (**A**) the soil concentration of organic pollutants, (**B**) mechanism of action, (**C**) exposure duration, and (**D**) the interaction between exposure duration and mechanism of action. In (**B**), open circles represent data points, and lower and upper boxes correspond to the 25th and 75th percentiles with the upper/lower whiskers extending from the box to the highest/smallest value at most 1.5 × interquartile range of the box. GABA = gamma‐aminobutyric acid gated chloride channel; nAChR = nicotinic acetylcholine receptor. Note that in (**D**), not all coefficients (sublevels) of the interaction between exposure duration and mechanism of action are shown as most of these sublevels did not have enough datapoints to assess interaction effects.

Through AIC model selection, a best model was identified which explained the variance significantly better than the reduced model (*χ* ^2^(12) = 36.254, *p* < 0.001). The publication identifier explained 20% of the total variance. Concentration of organic chemicals in soils had a small but significant positive effect on HSP70 gene expression (Table 5 and Figure 5A). There was also a significant interaction between exposure duration and mechanism of action (Figure 5D). However, exposure duration and mechanism of action alone did not significantly impact HSP70 gene expression (Figure 5B,C **)**.

**Table 5:** Statistics of the mixed‐effect model that best explained the earthworm HSP70 gene expression data (‘best model’)

	Dependent variable: Log2FC_MT_		
	Slope estimate	SD	*p* value
Constant	1.160	2.161	0.594
Concentration	0.384	0.140	0.008**
Exposure duration	−0.751	1.747	0.667
Mechanism of action			0.612
GABA	0.230	1.243	
nAChR	−0.876	2.207	
Nucleophilic	−1.168	1.118	
Plastoquinone binding	0.047	0.878	
Ryanodine receptor	0.047	0.878	
Succinate dehydrogenase	0.046	0.878	
Thyroid homeostasis	0.274	2.279	
Exposure duration:mechanism of action			<0.001***
nAChR	1.057	1.762	
Ryanodine receptor	3.968	1.946	
Succinate dehydrogenase	−0.364	1.834	
Observations	91		
Log likelihood	−102.40		
Akaike Information Criterion	234.80		
Bayesian Information Criterion	272.46		

Not all coefficients (sublevels) of the interaction between exposure duration and mechanism of action are listed as most of these sublevels did not have enough datapoints to assess interaction effects.

GABA = gamma‐aminobutyric acid gated chloride channel; Log2FCMT = log2 fold change of the gene expression of metallothionein (MT); nAChR = nicotinic acetylcholine receptor; SD = standard deviation.

**p < 0.01; ***p < 0.001.

## DISCUSSION

### Limitations of the meta‐analysis

This meta‐analysis assessed evidence in publications from peer‐reviewed scientific journals. Publishing negative results can be more difficult, and less motivating, than publishing positive outcomes. Hence, the results presented in our study may be biased towards positive effects. This could mean that some of the reported trends in the present study may be less evident in field applications than expected on the basis of meta‐analysis. Reporting bias is a recognized issue within meta‐analysis studies (Joober et al., 2012; Thornton & Lee, 2000). Publication bias can be mitigated by also including “grey literature” in any analysis (e.g., unpublished work, databases, PhD theses, etc.). However, such a systematic review approach went beyond the scope of the present study due to resource limitations and the time available for completion of our study.

Meta‐analyses, including in ecotoxicology, often use effect size as the measure for assessment (Isaksson, 2010; Ji et al., 2021). Because effects size measures, like Hedges' *g*, account for the error margins (e.g., standard deviation) around the means, such effect size metrics are considered more reliable measures of effects than, for example, simple fold changes of the mean. The calculation of effect size requires information not only on the means of the compared responses, but also on their standard deviation and sample size. In our study, not all identified publications reported these additional required details. Indeed, to have selected only those data that gave these three values for each data point would have resulted in the exclusion of roughly one‐third of all available data. Therefore in the present study, by using fold changes, we prioritized increasing the number of data points included in the meta‐analysis over the use of effect size as the more reliable measure of effect. Furthermore, fold changes are both more intuitive and easier to interpret than effect size measures, such as Hedges' *g*, as this is the basis of standard and widely used approaches within quantitative gene expression studies.

### MT expression in earthworms as a biomarker for metal exposure effects

The identification of transcriptional responses through the advanced use of RNAseq and other techniques will remain key to the initial identification of new gene expression biomarkers. Once identified, further mechanistic studies can assess the range of chemicals to which any such newly discovered biomarker will respond and the species in which such responses occur and can be reliably measured. As the volume of biomarkers studies increases, it becomes feasible to use evidence review methods to establish the robustness and application domains of developed methods. Such evidence assessments can provide an additional step beyond biomarker identification and mechanistic assessment, which is essential for their future diagnostic application. In the present study, we defined biomarker suitability to pollution assessment applications on the basis of five criteria relating to the responsiveness and robustness of the specific measure: 1) concentration dependency, 2) stability over time, 3) independence of species, 4) chemical specificity, and 5) indication for adverse effects on species or population level. The compliance of our focus biomarkers with the first three of these criteria were addressed by including these factors as explanatory variables within the statistical models used for data analysis. The last two criteria were assessed through literature studies as detailed below.

The meta‐analysis showed that MT expression in earthworms is strongly metal concentration dependent, consistent with the known mechanistic role of this protein in metal handling in a range of invertebrate species (Amiard et al., 2006; Dallinger et al., 2000). Although the modelling did not indicate that there was an interactive effect between metal and concentration, the concentration dependency was particularly clear for cadmium (Figure 4C). Earthworm species, exposure duration, and study type had little to no effect on expression level. However, modelling indicated that metal itself did have impact on MT gene expression. Compare to cadmium, copper had a negative impact on MT gene expression (see negative slope estimate in Table 4). Previous studies have also shown that the gene expression profile of earthworm MT is different under copper exposure to that of cadmium, with cadmium inducing far greater effects on gene expression than copper (Burgos et al., 2005; Galay‐Burgos et al., 2003; Spurgeon et al., 2004). These individual experimental conclusions are confirmed through the meta‐analysis (Figure 4C). Compared with cadmium, gold had a negative impact on MT gene expression, whereas silver and zinc were positively associated with MT expression, as for cadmium (note that that the different data points shown in the graphs are not all independent from each other but nested in “Publication,” therefore patterns—or in some cases the lack of—that can be observed through visual inspection do not always appear to match statistical results). For these latter three metals, there was comparatively little data available, meaning that further studies are needed to assess the robustness of these findings. Despite these indications of a metal‐specific response, the overall modelling outcomes showed no indication for an interaction effect between soil concentration and metal. However, the absence of an interaction effect could also reflect the data scarcity for metals other than cadmium, which for effects on MT expression is by far the most‐studied element.

Even though metal concentration was positively associated with earthworm MT gene expression, in roughly one‐third of cases MT was down‐regulated in exposed earthworms. This could reflect temporal fluctuations in gene regulation in response to an environmental stimulant, a response pattern that has been indicated from experimental studies (Nota et al., 2008; Simões et al., 2018). On the basis of the data provided, we were only able to link gene expression to total metal concentrations, which are not necessarily indicative of bioavailability (Lanno et al., 2004). In some cases of high total metal concentration, but low MT expression, test soil properties may be such that they limit metal bioavailability to the earthworms. This possibility was, however, difficult to assess because not all identified studies included information on major soil properties relevant to bioavailability (e.g., soil pH, organic matter content, cation exchange capacity, clay content).

In this meta‐analysis, we identified six studies that had measured MT gene expression in earthworms exposed to organic pollutants. In most of these studies, earthworm MT was not significantly or was only marginally up‐regulated (Nam et al., 2017; Novo et al., 2018; Yao et al., 2021). However, in a further study, MT in the earthworm *Eisenia fetida* was up‐regulated up to 30‐fold compared with controls after exposure to the antibiotic ciprofloxacin (Yang et al., 2020). Previous studies have also shown that under experimental laboratory conditions gene expression of MT in earthworms can be affected by nonchemical environmental variables like temperature. For example, exposure to cold strongly induced earthworm MT expression, especially when exposure took place in combination with copper exposure (Fisker et al., 2016). However, these effects may have been a response to cellular damage and may only occur under specific cold treatments that lead to intracellular ice formation. Indeed, under temperate field conditions, MT expression in earthworms was found to be only affected by soil metal concentrations and this response was not modified by season or temperature (Svendsen et al., 2007).

The median effect concentration (EC50) for earthworm reproduction for cadmium and copper has been reported to range between 0.17 and 2.6 mmol/kg soil (for cadmium) and 1.1 and 12.2 mmol/kg soil (for copper) depending on species, soil, and exposure duration (Criel et al., 2008; Hund‐Rinke & Simon, 2005; Scott‐Fordsmand et al., 2000; Spurgeon & Hopkin, 1995; Spurgeon et al., 1994, 2004; Spurgeon, Svendsen, et al., 2005). Based on the mixed‐effects models, these cadmium EC50s would induce between a 16‐ and 32‐fold change (i.e., a log2 fold change between 4 and 5) compared with nonexposed animals (Figure 4A) and exposure to a copper EC50 would induce between a 4‐ and 6‐fold change (i.e., log2 fold change of between 2 and 2.5). Thus, at concentrations likely to cause population effects MT up‐regulation is strongly indicative to cadmium exposure, but only weakly so for copper. Although up‐regulation of earthworm MT by environmental factors other than metal exposure has previously been observed under some specific conditions, such an effect does not seem to result in the same fold change effects as cadmium exposure. These findings from the meta‐analysis confirm that MT gene expression in earthworms is indeed a suitable marker for metal exposure effects, particularly for cadmium (Table 6).

**Table 6:** Evaluation of suitability of the two most studied species biomarkers to measure effects of chemical exposure

		Earthworm MT expression			
		Metals			Earthworm HSP70 expression
Criteria	Test method	All	Cd	Cu	Organics
Concentration‐dependent response	Models	+	++	?	–
Stable of time	Models	+	+	–	+
Universal to species group	Models	+	++	?	?
Specific to chemical group	Literature	+	–	–	–
Indicative for adverse effects	Literature	+	+	–	?

+ = some evidence/dependent on condition; ++ = strong evidence; – = no evidence; ? = not enough data; MT = metallothionein; HSP70 = heat shock protein 70.

### HSP70 expression in earthworms as biomarker for effects of exposure to organic pollutants

Heat shock proteins (HSPs) are a group of chaperones involved in cellular damage repair. Expression of HSPs in soil organisms can be induced by a range of environmental stress factors, including cold and heat shock and chemical exposure (Bahrndorff et al., 2009; Nadeau et al., 2001; Sørensen et al., 2010). As such, members of the HSP protein family are considered as a biomarker indicative of general stress. HSP70 was the biomarker most used to identify organic pollutant exposure and effect in earthworms. Multiple individual studies have reported HSP70 as a suitable biomarker for assessing exposure and effects of earthworms to different organic pollutants. This volume of research provided the ideal platform on which to test these individual recommendations to confirm the robustness and the chemical types for which this biomarker can be used. Contrary to the individual reports made in single studies, the meta‐analysis using all available data showed that the expression of HSP70 was not strongly dependent on organic chemical exposure concentration. Even at very high organic chemical concentrations, the HSP70 gene is only marginally up‐regulated in earthworms. Therefore, based on the analysis of the currently available information, HSP70 gene expression in earthworms does not meet the first and most important criteria for a successful biomarker. Therefore, earthworm HSP70 gene expression should not be recommended as a generalist and reliable marker for effects of organic chemical exposure (Table 5). That said, it has to be recognized that the current available data for HSP70 in earthworms for organic chemicals is limited. This reduces the analytical power. Earthworm HSP70 gene expression may well be a good indicator for exposure and effects for some individual chemicals. The present study also only measured HSP70 through transcriptional change, and did not consider whether protein measurement may be indicative of an expression response governed by translational change. However, based on the available data, our analysis does not support use of HSP70 gene expression in earthworms as a general indicator for organic chemical exposure effects.

### Future research needed on earthworm biomarkers

In the present study we sought to demonstrate how evidence review methods can be used to build on initial biomarker identification and mechanistic ecotoxicology studies to assess the robustness and specificity of proposed earthworm biomarkers. Metallothionein was confirmed as a reliable biomarker for cadmium. However, our meta‐analysis shows that MT gene expression in earthworms is not a reliable biomarker for copper exposure effects. This conclusion is in line with previous individual findings (Fisker et al., 2016; Galay‐Burgos et al., 2003; Homa et al., 2015; Spurgeon et al., 2004). In many agricultural regions, copper concentrations in top soils exceed risk quotient levels (Ballabio et al., 2018) and with copper‐based pesticides still in agricultural use (e.g., organic vineyards and soft fruit production) environmental copper concentrations in agricultural soils are expected to increase further (Droz et al., 2021). There is a need to develop a suitable biomarker indicative for copper that would allow the potential adverse effects of copper on soil invertebrate communities to be reliably monitored. In nematodes, studies have shown that the copper cytoplasmic metallochaperon protein (CUTC) plays a crucial role in protection from excess copper and that its gene expression may be used as a biomarker for this metal (Calafato et al., 2008). Recent studies have shown that also in earthworms, copper exposure strongly induces the expression of the CUTC gene and that this expression is stable over a 4‐day exposure period, making this gene a candidate biomarker for further studies (Hernadi, 2020).

Meta‐analysis showed that HSP70 gene expression in earthworms is not a reliable biomarker for effects of exposure to organic pollutants, either as a whole or for individual modes of action or compounds. Several studies have indicated that mRNA expression of CYP450 genes in springtails may be used as a biomarker for the exposure effects of organic pollutants (de Boer et al., 2011, 2013). The CYP450 enzyme are a family of functionally diverse catabolising enzymes, with some family members being involved in steroid synthesis and others playing a vital role in the phase I metabolism of xenobiotics. Earthworm species CYP450 gene families have not yet been mapped in detail. Although a draft genome of the earthworm *E. fetida* has been published (Paul et al., 2018), this genome has so far not been mined for CYP450 genes. Currently, only one CYP450 has been indicated to be responsive to xenobiotic exposure depending on dose (Li et al., 2018). A greater number of studies have measured the expression of genes for glutathione‐*S*‐transferases (GSTs), an enzyme family involved in phase II metabolism, as a potential biomarker for xenobiotic exposure and effects in earthworms (Hattab et al., 2015; Nam et al., 2017; Novo et al., 2018). One study has shown a GST family member to be up‐regulated in earthworms in a concentration‐dependent manner at the recommended application rates of a commonly applied herbicide (Hattab et al., 2015). Although preliminary, these studies of xenobiotic metabolizing enzymes indicate their potential as biomarkers for organic chemicals. To establish this potential, future studies should focus on the sequencing and functional annotation of genes in these enzyme families and the assessment of their expression under organic pollutant exposure.

## CONCLUSION

Through meta‐analysis, we have been able to confirm that MT gene expression in earthworms is a reliable and robust biomarker for the effects of exposure to cadmium, but not copper. The concentration‐response of MT expression under Cd exposure was independent of earthworm species. This indicates that for the use of this biomarker in, for example, biomonitoring of exposed populations, the choice of species would not affect the outcome of monitoring. This species independence makes this biomarker more universally applicable. Modelling indicated that gene expression profiles in earthworms exposed to cadmium in soils spiked in the laboratory were not different to that for earthworms exposed to metal polluted field soils. These findings indicate that results obtained from laboratory‐spiked soil exposure experiments are relevant for field conditions.

The HSP70 gene expression in earthworms is not found to be a reliable biomarker for organic pollutant exposure and effects. In the absence of a reliable biomarker for copper and organic xenobiotics, further work to identify potential suitable expression responses is needed. Such work should aim to identify and validate gene expression‐based biomarkers for copper and organic chemicals.

The present meta‐analysis was only applied to two biomarkers: MT gene expression in earthworms for metals and HSP70 expression in earthworms for organic chemicals. The focus on these two biomarkers was made based on the high availability of data on their responses to chemical exposure from across multiple studies, which allowed us to develop and test an approach for meta‐analysis for biomarker assessment and verification. The robustness of these biomarkers as indicators for chemical exposure effects was variable. We recognize that there are many other proposed gene expression‐based biomarkers that may be more (or less) suitable as indicators of chemical exposure effects in soil invertebrates. In cases where data on the responses of these different biomarkers to chemical exposure are available, then it would be possible to undertake meta‐analysis studies, similar to our study, to assess the robustness and application domain of these different proposed measures. All raw data and R scripts used for the present analysis are openly available (as supplementary information and under Creative Commons Attribution 4.0 at 10.5281/zenodo.5145029). We believe that such quantitative assessment and validation of robustness is a prerequisite for the use of gene expression biomarkers in soil ecotoxicological experiments and in biomonitoring. Therefore, we strongly encourage colleagues in the field to quantitatively test other biomarkers for robustness using meta‐analysis methods.

## Supporting Information

The Supporting Information is available on the Wiley Online Library at https://doi.org/10.1002/etc.5402.

## Author Contribution Statement

**Ellie Martell**: Conceptualization; Project administration; Writing—review & editing. **David J. Spurgeon**: Conceptualization; Funding acquisition; Methodology; Formal analysis; Investigation; Project administration; Supervision; Writing—review & editing. **Elmer Swart**: Conceptualization; Formal analysis; Investigation; Methodology; Data curation; Writing—original draft. **Claus Svendsen**: Funding acquisition; Conceptualization; Project administration; Writing—review & editing.

###  

This article has earned both an Open Data and an Open Materials badge for making publicly available the digitally‐shareable data necessary to reproduce the reported results. The data is available at https://doi.org/10.5281/zenodo.5145029. Learn more about the Open Practices badges from the Center for Open Science: https://osf.io/tvyxz/wiki.

## Supporting information

This article includes online‐only Supporting Information.

Supporting information.

Supporting information.

## Data Availability

The data and R scripts that support the findings of the present study are openly available under Creative Commons Attribution 4.0 at 10.5281/zenodo.5145029. We actively invite other researchers to use the data and build the database further with gene expression data for other biomarkers.

## References

[etc5402-bib-0001] Amiard, J. C. , Amiard‐Triquet, C. , Barka, S. , Pellerin, J. , & Rainbow, P. S. (2006). Metallothioneins in aquatic invertebrates: Their role in metal detoxification and their use as biomarkers. Aquatic Toxicology, 76(2), 160–202. 10.1016/j.aquatox.2005.08.015 16289342

[etc5402-bib-0002] Amiard‐Triquet, C. , & Amiard, J. C. (2013). Ecological biomarkers: Indicators of ecotoxicological effects. *53*, 1689–1699.

[etc5402-bib-0003] Bahrndorff, S. , Mariën, J. , Loeschcke, V. , & Ellers, J. (2009). Dynamics of heat‐induced thermal stress resistance and hsp70 expression in the springtail, *Orchesella cincta* . Functional Ecology, 23(2), 233–239. 10.1111/j.1365-2435.2009.01541.x

[etc5402-bib-0004] Ballabio, C. , Panagos, P. , Lugato, E. , Huang, J.‐H. , Orgiazzi, A. , Jones, A. , Fernández‐Ugalde, O. , Borrelli, P. , & Montanarella, L. (2018). Copper distribution in European topsoils: An assessment based on {LUCAS} soil survey. Science of the Total Environment, 636, 282–298. 10.1016/j.scitotenv.2018.04.268 29709848

[etc5402-bib-0005] Barton, K. (2020). MuMIn: Multi‐Model Inference. R package version 1.43.17. https://cran.r-project.org/package=MuMIn

[etc5402-bib-0008] de Boer, M. E. , Berg, S. , Timmermans, M. J. T. N. , den Dunnen, J. T. , van Straalen, N. M. , Ellers, J. , & Roelofs, D. (2011). High throughput nano‐liter RT‐qPCR to classify soil contamination using a soil arthropod. BMC Molecular Biology, 12, 11. 10.1186/1471-2199-12-11 21362169 PMC3060125

[etc5402-bib-0009] De Boer, M. E. , Ellers, J. , Van Gestel, C. A. M. , Den Dunnen, J. T. , Van Straalen, N. M. , & Roelofs, D. (2013). Transcriptional responses indicate attenuated oxidative stress in the springtail *Folsomia candida* exposed to mixtures of cadmium and phenanthrene. Ecotoxicology, 22(4), 619–631. 10.1007/s10646-013-1053-1 23483327

[etc5402-bib-0010] Brack, W. , Ait‐Aissa, S. , Burgess, R. M. , Busch, W. , Creusot, N. , Di Paolo, C. , Escher, B. I. , Mark Hewitt, L. , Hilscherova, K. , Hollender, J. , Hollert, H. , Jonker, W. , Kool, J. , Lamoree, M. , Muschket, M. , Neumann, S. , Rostkowski, P. , Ruttkies, C. , Schollee, J. , … Krauss, M. (2016). Effect‐directed analysis supporting monitoring of aquatic environments—An in‐depth overview. Science of the Total Environment, 544, 1073–1118. 10.1016/j.scitotenv.2015.11.102 26779957

[etc5402-bib-0011] Brulle, F. , Mitta, G. , Leroux, R. , Lemière, S. , Leprêtre, A. , & Vandenbulcke, F. (2007). The strong induction of metallothionein gene following cadmium exposure transiently affects the expression of many genes in *Eisenia fetida*: A trade‐off mechanism? Comparative Biochemistry and Physiology—C Toxicology and Pharmacology, 144(4), 334–341. 10.1016/j.cbpc.2006.10.007 17150412

[etc5402-bib-0012] Burgos, M. G. , Winters, C. , Stürzenbaum, S. R. , Randerson, P. F. , Kille, P. , & Morgan, A. J. (2005). Cu and Cd effects on the earthworm *Lumbricus rubellus* in the laboratory: Multivariate statistical analysis of relationships between exposure, biomarkers, and ecologically relevant parameters. Environmental Science and Technology, 39(6), 1757–1763. 10.1021/es049174x 15819235

[etc5402-bib-0013] Burns, E. E. , & Boxall, A. B. A. (2018). Microplastics in the aquatic environment: Evidence for or against adverse impacts and major knowledge gaps. Environmental Toxicology and Chemistry, 37(11), 2776–2796. 10.1002/etc.4268 30328173

[etc5402-bib-0014] Calafato, S. , Swain, S. , Hughes, S. , Kille, P. , & Stürzenbaum, S. R. (2008). Knock down of caenorhabditis elegans cutc‐1 exacerbates the sensitivity toward high levels of copper. Toxicological Sciences, 106(2), 384–391. 10.1093/toxsci/kfn180 18723824

[etc5402-bib-0015] Cedergreen, N. (2014). Quantifying synergy: A systematic review of mixture toxicity studies within environmental toxicology. PLoS One, 9(5), e96580. 10.1371/journal.pone.0096580 24794244 PMC4008607

[etc5402-bib-0016] Chao, H. Z. , Sun, M. M. , Wu, Y. L. , Xia, R. , Yuan, S. J. , & Hu, F. (2022). Quantitative relationship between earthworms' sensitivity to organic pollutants and the contaminants' degradation in soil: A meta‐analysis. Journal of Hazardous Materials, 429, 128286. 10.1016/j.jhazmat.2022.128286 35086042

[etc5402-bib-0017] Colin, N. , Porte, C. , Fernandes, D. , Barata, C. , Padrós, F. , Carrassón, M. , Monroy, M. , Cano‐Rocabayera, O. , de Sostoa, A. , Piña, B. , & Maceda‐Veiga, A. (2016). Ecological relevance of biomarkers in monitoring studies of macro‐invertebrates and fish in Mediterranean rivers. Science of the Total Environment, 540, 307–323. 10.1016/j.scitotenv.2015.06.099 26148426

[etc5402-bib-0018] Collins, A. , Coughlin, D. , Miller, J. , & Kirk, S. (2015). The production of quick scoping reviews and rapid evidence assessments: A how to guide. https://www.gov.uk/government/publications/the-production-of-quick-scoping-reviews-and-rapid-evidence-assessments

[etc5402-bib-0019] Criel, P. , Lock, K. , Van Eeckhout, H. , Oorts, K. , Smolders, E. , & Janssen, C. R. (2008). Influence of soil properties on copper toxicity for two soil invertebrates. Environmental Toxicology and Chemistry, 27(8), 1748–1755. 10.1897/07-545.1 18290689

[etc5402-bib-0020] Dallinger, R. , Berger, B. , Gruber, C. , Hunziker, P. , & Stürzenbaum, S. (2000). Metallothioneins in terrestrial invertebrates: Structural aspects, biological significance and implications for their use as biomarkers. Cellular and Molecular Biology, 46(2), 331–46. 10774923

[etc5402-bib-0021] Droz, B. , Payraudeau, S. , Rodríguez Martín, J. A. , Tóth, G. , Panagos, P. , Montanarella, L. , Borrelli, P. , & Imfeld, G. (2021). Copper content and export in European vineyard soils influenced by climate and soil properties. Environmental Science and Technology, 55(11), 7327–7334. 10.1021/acs.est.0c02093 34009978

[etc5402-bib-0022] Eom, I. C. , Rast, C. , Veber, A. M. , & Vasseur, P. (2007). Ecotoxicity of a polycyclic aromatic hydrocarbon (PAH)‐contaminated soil. Ecotoxicology and Environmental Safety, 67(2), 190–205. 10.1016/j.ecoenv.2006.12.020 17382389

[etc5402-bib-0023] Fisker, K. V. , Holmstrup, M. , & Sørensen, J. G. (2016). Freezing of body fluids induces metallothionein gene expression in earthworms (*Dendrobaena octaedra*). Comparative Biochemistry and Physiology Part‐C Toxicology and Pharmacology, 179, 44–48. 10.1016/j.cbpc.2015.08.008 26325206

[etc5402-bib-0024] Forbes, V. E. , Palmqvist, A. , & Bach, L. (2006). The use and misuse of biomarkers in ecotoxicology. Environmental Toxicology and Chemistry, 25(1), 272–280. 10.1897/05-257R.1 16494252

[etc5402-bib-0025] Galay‐Burgos, M. , Spurgeon, D. J. , Weeks, J. M. , Stürzenbaum, S. R. , Morgan, A. J. , & Kille, P. (2003). Developing a new method for soil pollution monitoring using molecular genetic biomarkers. Biomarkers, 8(4/3), 229–239. 10.1080/354750031000138685 12944175

[etc5402-bib-0026] Gong, P. , & Perkins, E. J. (2016). Earthworm toxicogenomics: A renewed genome‐wide quest for novel biomarkers and mechanistic insights. Applied Soil Ecology, 104, 12–24. 10.1016/j.apsoil.2015.11.005

[etc5402-bib-0028] Hattab, S. , Boughattas, I. , Boussetta, H. , Viarengo, A. , Banni, M. , & Sforzini, S. (2015). Transcriptional expression levels and biochemical markers of oxidative stress in the earthworm *Eisenia andrei* after exposure to 2,4‐dichlorophenoxyacetic acid (2,4‐D). Ecotoxicology and Environmental Safety, 122, 76–82. 10.1016/j.ecoenv.2015.07.014 26210610

[etc5402-bib-0029] Hernadi, S. B. (2020). Earthworm system immunity and its modulation by nanoparticles. Cardiff University.

[etc5402-bib-0030] Homa, J. , Rorat, A. , Kruk, J. , Cocquerelle, C. , Plytycz, B. , & Vandenbulcke, F. (2015). Dermal exposure of *Eisenia andrei* earthworms: Effects of heavy metals on metallothionein and phytochelatin synthase gene expressions in coelomocytes. Environmental Toxicology and Chemistry, 34(6), 1397–1404. 10.1002/etc.2944 25693738

[etc5402-bib-0031] Hund‐Rinke, K. , & Simon, M. (2005). Terrestrial ecotoxicity of eight chemicals in a systematic approach. Journal of Soils and Sediments, 5(1), 59–65. 10.1065/jss2004.10.123

[etc5402-bib-0032] Isaksson, C. (2010). Pollution and its impact on wild animals: A meta‐analysis on oxidative stress. EcoHealth, 7(3), 342–350. 10.1007/s10393-010-0345-7 20865439

[etc5402-bib-0033] Ji, Z. , Huang, Y. , Feng, Y. , Johansen, A. , Xue, J. , Tremblay, L. A. , & Li, Z. (2021). Effects of pristine microplastics and nanoplastics on soil invertebrates: A systematic review and meta‐analysis of available data. *Science of the Total Environment*, *788*. 10.1016/j.scitotenv.2021.147784 34029821

[etc5402-bib-0034] Joober, R. , Schmitz, N. , Annable, L. , & Boksa, P. (2012). Publication bias: What are the challenges and can they be overcome? Journal of Psychiatry and Neuroscience, 37(3), 149–152. 10.1503/jpn.120065 22515987 PMC3341407

[etc5402-bib-0035] Kortenkamp, A. , & Faust, M. (2018). Regulate to reduce chemical mixture risk. Science, 361(6399), 224–226. 10.1126/science.aat9219 30026211

[etc5402-bib-0036] Kuznetsova, A. , Brockhoff, P. B. , & Christensen, R. H. B. (2017). lmerTest Package: Tests in linear mixed effects models. Journal of Statistical Software, 82(13), 1–26. 10.18637/jss.v082.i13

[etc5402-bib-0037] Lanno, R. , Wells, J. , Conder, J. , Bradham, K. , & Basta, N. (2004). The bioavailability of chemicals in soil for earthworms. Ecotoxicology and Environmental Safety, 57(1), 39–47. 10.1016/j.ecoenv.2003.08.014 14659365

[etc5402-bib-0038] Li, Y. , Zhao, C. , Lu, X. , Ai, X. , & Qiu, J. (2018). Identification of a cytochrome P450 gene in the earthworm *Eisenia fetida* and its mRNA expression under enrofloxacin stress. Ecotoxicology and Environmental Safety, 150(December 2017), 70–75. 10.1016/j.ecoenv.2017.12.020 29268117

[etc5402-bib-0039] McCarthy, J. F. , & Shugart, L. R. (1990). J. F. McCarthy & L. R. Shugart (Eds.), Biomarkers of environmental contamination. Boca Raton, FL: Lewis.

[etc5402-bib-0040] Mirmonsef, H. , Hornum, H. D. , Jensen, J. , & Holmstrup, M. (2017). Effects of an aged copper contamination on distribution of earthworms, reproduction and cocoon hatchability. Ecotoxicology and Environmental Safety, 135(June 2016), 267–275. 10.1016/j.ecoenv.2016.10.012 27750094

[etc5402-bib-0041] Nadeau, D. , Corneau, S. , Plante, I. , Morrow, G. , & Tanguay, R. M. (2001). Evaluation for Hsp70 as a biomarker of effect of pollutants on the earthworm *Lumbricus terrestris* . Cell Stress and Chaperones, 6(2), 153–163. 10.1379/1466-1268(2001)006<0153:EFHAAB>2.0.CO;2 11599577 PMC434393

[etc5402-bib-0042] Nam, T. H. , Kim, L. , Jeon, H. J. , Kim, K. , Ok, Y. S. , Choi, S. D. , & Lee, S. E. (2017). Biomarkers indicate mixture toxicities of fluorene and phenanthrene with endosulfan toward earthworm (*Eisenia fetida*). Environmental Geochemistry and Health, 39(2), 307–317. 10.1007/s10653-016-9876-3 27696228

[etc5402-bib-0043] Nota, B. , Timmermans, M. J. T. N. , Franken, O. , Montagne‐Wajer, K. , Mariën, J. , De Boer, M. E. , De Boer, T. E. , Ylstra, B. , Van Straalen, N. M. , & Roelofs, D. (2008). Gene expression analysis of collembola in cadmium containing soil. Environmental Science and Technology, 42(21), 8152–8157. 10.1021/es801472r 19031917

[etc5402-bib-0044] Notter, D. , Mitrano, D. M. , & Nowack, B. (2014). Are nanosized or dissolved metals more toxic? A meta‐analysis. Environmental Toxicology and Chemistry, 33, 2733–2739. 10.1002/etc.2732 25158308

[etc5402-bib-0045] Novais, S. C. , de Coen, W. , & Amorim, M. J. B. (2012). Gene expression responses linked to reproduction effect concentrations (EC10,20,50,90) of dimethoate, atrazine and carbendazim, in enchytraeus albidus. PLoS One , 7(4),. 10.1371/journal.pone.0036068 PMC3338630 22558331

[etc5402-bib-0046] Novo, M. , Verdú, I. , Trigo, D. , & Martínez‐Guitarte, J. L. (2018). Endocrine disruptors in soil: Effects of bisphenol A on gene expression of the earthworm *Eisenia fetida* . Ecotoxicology and Environmental Safety, 150(January), 159–167. 10.1016/j.ecoenv.2017.12.030 29275183

[etc5402-bib-0047] Paul, S. , Arumugaperumal, A. , Rathy, R. , Ponesakki, V. , Arunachalam, P. , & Sivasubramaniam, S. (2018). Data on genome annotation and analysis of earthworm *Eisenia fetida* . Data in Brief, 20, 525–534. 10.1016/j.dib.2018.08.067 30191166 PMC6126081

[etc5402-bib-0048] Pérès, G. , Vandenbulcke, F. , Guernion, M. , Hedde, M. , Beguiristain, T. , Douay, F. , Houot, S. , Piron, D. , Richard, A. , Bispo, A. , Grand, C. , Galsomies, L. , & Cluzeau, D. (2011). Earthworm indicators as tools for soil monitoring, characterization and risk assessment. An example from the National Bioindicator Programme (France). Pedobiologia (Jena) , 54(Suppl),. 10.1016/j.pedobi.2011.09.015

[etc5402-bib-0049] Pelosi, C. , Joimel, S. , & Makowski, D. (2013). Searching for a more sensitive earthworm species to be used in pesticide homologation tests—A meta‐analysis. Chemosphere, 90, 895–900. 10.1016/j.chemosphere.2012.09.034 23084259

[etc5402-bib-0050] Richard, A. M. , Judson, R. S. , Houck, K. A. , Grulke, C. M. , Volarath, P. , Thillainadarajah, I. , Yang, C. , Rathman, J. , Martin, M. T. , Wambaugh, J. F. , Knudsen, T. B. , Kancherla, J. , Mansouri, K. , Patlewicz, G. , Williams, A. J. , Little, S. B. , Crofton, K. M. , & Thomas, R. S. (2016). ToxCast chemical landscape: Paving the road to 21st century toxicology. Chemical Research in Toxicology, 29(8), 1225–1251. 10.1021/acs.chemrestox.6b00135 27367298

[etc5402-bib-0051] Roelofs, D. , de Boer, M. , Agamennone, V. , Bouchier, P. , Legler, J. , & van Straalen, N. (2012). Functional environmental genomics of a municipal landfill soil. Frontiers in Genetics, 3, 85. 10.3389/fgene.2012.00085. 22623925 PMC3353140

[etc5402-bib-0052] Roelofs, D. , Janssens, T. K. S. , Timmermans, M. J. T. N. , Nota, B. , MariËn, J. , Bochdanovits, Z. , Ylstra, B. , & Van Straalen, N. M. (2009). Adaptive differences in gene expression associated with heavy metal tolerance in the soil arthropod *Orchesella cincta* . Molecular Ecology, 18(15), 3227–3239. 10.1111/j.1365-294X.2009.04261.x 19566677

[etc5402-bib-0053] Rohatgi, A. (2020). WebPlotDigitizer v4.4. Retreived February 1, 2021, from: https://automeris.io/WebPlotDigitizer

[etc5402-bib-0054] Scott‐Fordsmand, J. J. , Weeks, J. M. , & Hopkin, S. P. (2000). Importance of contamination history for understanding toxicity of copper to earthworm *Eisenia fetica* (Oligochaeta: Annelida), using neutral‐red retention assay. Environmental Toxicology and Chemistry, 19(7), 1774–1780. 10.1002/etc.5620190710

[etc5402-bib-0055] Shahid, M. (2021). Effect of soil amendments on trace element‐mediated oxidative stress in plants: Meta‐analysis and mechanistic interpretations. Journal of Hazardous Materials, 407, 124881. 10.1016/j.jhazmat.2020.124881 33360193

[etc5402-bib-0056] Simões, T. , Novais, S. C. , Natal‐da‐Luz, T. , Devreese, B. , de Boer, T. , Roelofs, D. , Sousa, J. P. , van Straalen, N. M. , & Lemos, M. F. L. (2018). An integrative omics approach to unravel toxicity mechanisms of environmental chemicals: Effects of a formulated herbicide. Scientific Reports, 8, 11376. 10.1038/s41598-018-29662-6 30054531 PMC6063884

[etc5402-bib-0057] Sørensen, J. G. , Heckmann, L. H. , & Holmstrup, M. (2010). Temporal gene expression profiles in a palaearctic springtail as induced by desiccation, cold exposure and during recovery. Functional Ecology, 24(4), 838–846. 10.1111/j.1365-2435.2010.01687.x

[etc5402-bib-0058] Spurgeon, D. J. , & Hopkin, S. P. (1995). Extrapolation of the laboratory‐based OECD earthworm toxicity test to metal‐contaminated field sites. Ecotoxicology, 4(3), 190–205. 10.1007/BF00116481 24197704

[etc5402-bib-0059] Spurgeon, D. J. , & Hopkin, S. P. (1996). The effects of metal contamination on earthworm populations around a smelting works: Quantifying species effects. Applied Soil Ecology, 4(2), 147–160. 10.1016/0929-1393(96)00109-6

[etc5402-bib-0060] Spurgeon, D. J. , Hopkin, S. P. , & Jones, D. T. (1994). Effects of cadmium, copper, lead and zinc on growth, reproduction and survival of the earthworm *Eisenia fetida* (Savigny): Assessing the environmental impact of point‐source metal contamination in terrestrial ecosystems. Environmental Pollution, 84(2), 123–130. 10.1016/0269-7491(94)90094-9 15091707

[etc5402-bib-0061] Spurgeon, D. J. , Ricketts, H. , Svendsen, C. , John Morgan, A. , & Kille, P. (2005). Hierarchical responses of soil invertebrates (earthworms) to toxic metal stress. Environmental Science & Technology, 39(14), 5327–5334. 10.1021/es050033k 16082963

[etc5402-bib-0062] Spurgeon, D. J. , Stürzenbaum, S. R. , Svendsen, C. , Hankard, P. K. , Morgan, A. J. , Weeks, J. M. , & Kille, P. (2004). Toxicological, cellular and gene expression responses in earthworms exposed to copper and cadmium. Comparative Biochemistry and Physiology—C Toxicology and Pharmacology, 138(1), 11–21. 10.1016/j.cca.2004.04.003 15313442

[etc5402-bib-0063] Spurgeon, D. J. , Svendsen, C. , Lister, L. J. , Hankard, P. K. , & Kille, P. (2005). Earthworm responses to Cd and Cu under fluctuating environmental conditions: A comparison with results from laboratory exposures. Environmental Pollution, 136(3), 443–452. 10.1016/j.envpol.2005.01.013 15862398

[etc5402-bib-0064] Van Straalen, N. M. , & Roelofs, D. (2008). Genomics technology for assessing soil pollution. Journal of Biology, 7, 19. 10.1186/jbiol80 18638358 PMC2776391

[etc5402-bib-0065] Stürzenbaum, S. R. , Kille, P. , & Morgan, A. J. (1998). The identification, cloning and characterization of earthworm metallothionein. FEBS Letters, 431(3), 437–442. 10.1016/S0014-5793(98)00809-6 9714559

[etc5402-bib-0066] Svendsen, C. , Hankard, P. K. , Lister, L. J. , Fishwick, S. K. , Jonker, M. J. , & Spurgeon, D. J. (2007). Effect of temperature and season on reproduction, neutral red retention and metallothionein responses of earthworms exposed to metals in field soils. Environmental Pollution, 147(1), 83–93. 10.1016/j.envpol.2006.08.012 17045713

[etc5402-bib-0067] Svendsen, C. , Owen, J. , Kille, P. , Wren, J. , Jonker, M. J. , Headley, B. A. , Morgan, A. J. , Blaxter, M. , Stürzenbaum, S. R. , Hankard, P. K. , Lister, L. J. , & Spurgeon, D. J. (2008). Comparative transcriptomic responses to chronic cadmium, fluoranthene, and atrazine exposure in Lumbricus rubellus. Environmental Science and Technology, 42(11), 4208–4214. 10.1021/es702745d 18589989

[etc5402-bib-0068] Swart, E. , Martell, E. , Svendsen, C. , & Spurgeon, D. J. (2022). *Soil ecotoxicology needs robust biomarkers – an meta‐analysis approach to test the robustness of gene expression‐based biomarkers for measuring chemical exposure effects in soil invertebrates* (v1.0). 10.5281/zenodo.5145029 PMC9543370 35698918

[etc5402-bib-0070] Thornton, A. , & Lee, P. (2000). Publication bias in meta‐analysis: Its causes and consequences. Journal of Clinical Epidemiology, 53(2), 207–216. 10.1016/S0895-4356(99)00161-4 10729693

[etc5402-bib-0071] Timmermans, M. J. T. N. , Ellers, J. , Roelofs, D. , & Van Straalen, N. M. (2005). Metallothionein mRNA expression and cadmium tolerance in metal‐stressed and reference populations of the springtail Orchesella cincta. Ecotoxicology, 14(7), 727–739. 10.1007/s10646-005-0020-x 16160751

[etc5402-bib-0074] Yang, X. , Li, Y. , & Wang, X. (2020). Effects of ciprofloxacin exposure on the earthworm *Eisenia fetida* . Environmental Pollution, 262, 114287. 10.1016/j.envpol.2020.114287 32146370

[etc5402-bib-0075] Yao, X. , Qiao, Z. , Zhang, F. , Liu, X. , Du, Q. , Zhang, J. , Li, X. , & Jiang, X. (2021). Effects of a novel fungicide benzovindiflupyr in *Eisenia fetida*: Evaluation through different levels of biological organization. Environmental Pollution, 271, 116336. 10.1016/j.envpol.2020.116336 33370611

