# Supplementary material for: Soil Ecotoxicology Needs Robust Biomarkers: A Meta‐Analysis Approach to Test the Robustness of Gene Expression‐Based Biomarkers for Measuring Chemical Exposure Effects in Soil Invertebrates
Source: Environ Toxicol Chem. 2022 Aug 3;41(9):2124–38. doi: 10.1002/etc.5402 (PMC9543370; doi:10.1002/etc.5402)
Supplement: Supplementary file 1 — Supporting information. [file ETC-41-2124-s001.docx]

**SUPPLEMENTARY INFORMATION**

**Table S1:** Variables included in the linear mixed effect models

| **Variable** | **Measure** | **Type** | **Description** |
| --- | --- | --- | --- |
| Log2FC_MT_ | Continuous | Response | Log2 fold change between earthworm MT expression of treatment compared to control |
| Log2FC_HSP70_ | Continuous | Response | Log2 fold change between earthworm HSP70 expression of treatment compared to control |
| Species | Factor | Fixed effect | Earthworm species: *Eisenia fetida*, *Eisenia* *andrei*, *Lumbricus rubellus*, *Lumbricus terrestris*, *Aporrectodea caliginosa* |
| Exposure duration | Continuous | Fixed effect | Log10 transformed exposure duration in days |
| Concentration | Continuous | Fixed effect | Log10 transformed concentration of chemicals in µmol per kg soil (moles were used to be able to compare different chemical by number of molecules instead of mass) |
| Metal | Factor | Fixed effect | Metal: cadmium, zinc, copper, silver, gold |
| Organic mechanism of action | Factor | Fixed effect | Mechanism of action of the organic chemical |
| Study type | Factor | Fixed effect | ‘Spiked experimental studies’ or ‘Polluted field soil experimental studies’ |
| Publication | Factor | Random effect | Publication identifier |

**Table S2**: results of the mixed effect models to investigate which interaction effects should be included in the global models

| **Interaction tested** | **p-value** | **Included in global model?** |
| --- | --- | --- |
| *MT gene expression in earthworms* | | |
| Concentration:Exposure duration | 0.202 | No |
| Concentration:Species | 0.078 | No |
| Concentration:Metal | 0.403 | No |
| Concentration:Study type | 0.758 | No |
| Exposure duration:Species | 0.369 | No |
| Exposure duration:Metal | <0.001 *** | Yes |
| Exposure duration:Study type | 0.087 | No |
| *HSP70 gene expression in earthworms* | | |
| Concentration:Exposure duration | 0.038 * | Yes |
| Concentration:Mechanism of action | 0.825 | No |
| Exposure duration:Mechanism of action | 0.001** | Yes |

* p<0.05, ** p<0.01, *** p<0.001.
